# Increase in the proportion of *Plasmodium falciparum* with *kelch13* C580Y mutation and decline in *pfcrt* and *pfmdr1* mutant alleles in Papua New Guinea

**DOI:** 10.1186/s12936-021-03933-6

**Published:** 2021-10-19

**Authors:** Naoko Yoshida, Masato Yamauchi, Ryosuke Morikawa, Francis Hombhanje, Toshihiro Mita

**Affiliations:** 1 Department of Tropical Medicine and Parasitology, Faculty of Medicine, Juntendo University, 2-1-1 Hongo, Bunkyo-ku, Tokyo, 113-0033 Japan; 2 Centre for Health Research & Diagnostics, Divine Word University, P.O. Box 483, Madang, Papua New Guinea

**Keywords:** *Plasmodium falciparum*, Artemisinin, Chloroquine, Lumefantrine, Resistance, *kelch13*, *pfcrt*, *pfmdr1*, C580Y, Papua New Guinea

## Abstract

**Background:**

The C580Y mutation in the *Plasmodium falciparum kelch13* gene is the most commonly observed variant in artemisinin-resistant isolates in the Greater Mekong Subregion (GMS). Until 2017, it had not been identified outside the GMS, except for Guyana/Amazonia. In 2017, three parasites carrying the C580Y mutation were identified in Papua New Guinea (PNG). As the C580Y allele rapidly spread in the GMS, there is concern that this mutant is now spreading in PNG.

**Methods:**

In 2020, a cross-sectional survey was conducted at two clinics in Wewak, PNG. Symptomatic patients infected with *P. falciparum* were treated with artemether plus lumefantrine following a national treatment policy. Blood samples were obtained before treatment, and polymorphisms in *kelch13*, *pfcrt*, and *pfmdr1* were determined. Parasite positivity was examined on day 3. The results were compared with those of previous studies conducted in 2002, 2003, and 2016–2018.

**Results:**

A total of 94 patients were included in this analysis. The proportion of C580Y was significantly increased (2.2% in 2017, 5.7% in 2018, and 6.4% in 2020; p = 4.2 × 10^–3^). A significant upward trend was observed in the wild-type proportion for *pfcrt* (1.9% in 2016 to 46.7% in 2020; p = 8.9 × 10^–16^) and *pfmdr1* (59.5% in 2016 to 91.4% in 2020; p = 2.3 × 10^–6^). Among 27 patients successfully followed on day 3, including three with C580Y infections, none showed positive parasitaemia.

**Conclusions:**

Under the conditions of significant increases in *pfcrt* K76 and *pfmdr1* N86 alleles in PNG, the increase in *kelch13* C580Y mutants may be a warning indicator of the emergence of parasites resistant to the currently used first-line treatment regimen of artemether plus lumefantrine. Therefore, nationwide surveillance of molecular markers for drug resistance and assessment of its therapeutic effects are important.

**Supplementary Information:**

The online version contains supplementary material available at 10.1186/s12936-021-03933-6.

## Background

Artemisinin (ART)-based combination therapy (ACT) is a widely used first-line treatment for uncomplicated malaria. Malaria deaths have markedly decreased since the introduction of the treatment in the early 2000s [1]. However, the emergence of ART-resistant *Plasmodium falciparum* was first reported in the Greater Mekong Subregion (GMS) in 2006 [2]. Since then, ART-resistant parasites have rapidly spread in the region, partly because of the emergence of resistance to partner drug(s) of ACT [3, 4]. Therefore, the emergence and spread of ART-resistant parasites outside the GMS has become a global concern.

Propeller polymorphisms of the *kelch13* are useful molecular markers for monitoring the emergence and spread of ART resistance [3, 5]. To date, ten non-synonymous mutations in *kelch13* have been validated as polymorphisms for ART resistance. These include F446I, N458Y, M476I, Y493H, R539T, I543T, P553L, R561H, P574L, and C580Y [6]. In particular, C580Y has gradually outcompeted the other mutations and become dominant in some parts of the GMR region [7, 8]. C580Y is considered the most useful molecular marker for tracing the spread of ART resistance in GMS. However, outside the GMS region, this mutation has been detected only in Guyana/Amazonia [9, 10] and, more recently, in Papua New Guinea (PNG) [11].

In PNG, ACT was officially introduced as the first-line treatment regimen for uncomplicated malaria in 2010. Until then, chloroquine plus sulfadoxine/pyrimethamine was used. This therapy was subsequently replaced with artemether plus lumefantrine (AL). In 2017, three *P. falciparum* parasites harbouring C580Y were identified in Wewak, East Sepik. Population-genetic analysis using whole-genome and haplotypes of *kelch13* flanking microsatellite markers suggested that the parasites harbouring C580Y in PNG did not migrate from Southeast Asia. Rather, they had emerged independently from another region in New Guinea [11]. Considering the aggressive increase in the C580Y harbouring parasites in GMS region, there is a growing concern that a similar phenomenon may occur in PNG. Therefore, it is essential to assess whether the population of parasites harbouring C580Y has increased in the total parasite population in PNG.

In addition to the issue of ART resistance, previous ex vivo drug study also found that most *P. falciparum* parasites were resistant to chloroquine despite the discontinuation of chloroquine use in the early 2010s [12]. This is contrary to the observations in many African countries where chloroquine susceptibility recovered years after discontinuation [13–15].

To evaluate whether the parasites harbouring the C580Y mutation have increased since the first emergence and whether chloroquine resistance persists, a molecular epidemiological study was performed in 2020 in Wewak, East Sepik. The results were analysed with previous results obtained in 2002, 2003, and 2016–2018 [11, 12, 16, 17], which were conducted with the same design, target populations, and site as the present study. The results showed a significant increase in C580Y proportion and potential recovery of chloroquine susceptibility.

## Methods

### Study design and site

This study was conducted at two clinics (Wirui Urban and Town) in January and February 2020 in Wewak District, East Sepik Province, PNG [12]. The study area comprises a lowland swamp along the coast. High transmission rates of malaria occur throughout the year, with seasonal fluctuations [18]. Four *Plasmodium* species (*P. falciparum, Plasmodium vivax, Plasmodium ovale,* and *Plasmodium malariae*) were observed in this region, with *P*. *falciparum* predominant.

Ethical approval for the study was obtained from the Medical Research Ethical Committee of Juntendo University (No. 2017070) and the Medical Research Advisory Committee of the PNG National Department of Health (MRAC No.16.41).

### Patients and blood collection

In both clinics, patients with suspected malarial symptoms were screened using the Rapid Diagnosis Test (RDT) (Carestart™ Malaria HRP2/pLDH COMBO, Access Bio Inc., NJ, USA). Patients > 1 year of age with *Plasmodium*-positive results were recruited for the study and were enrolled after obtaining informed consent from the patients or their guardians. Patients who met the criteria for severe malaria and pregnant women in 1st trimester were not included. Blood samples (100 µl) were obtained through finger prick and transferred onto ET31CHR chromatography filter paper (Whatman Limited, Kent, UK). After drying at room temperature, the samples were separated in a plastic bag and stored at − 20 °C. Thick and thin blood smears were prepared and stained with 2% Giemsa for 45 min for parasite counting. Parasitaemia was determined by counting 1000 erythrocytes on a thin smear or 200 leukocytes on a thick smear (parasitaemia < 0.1%). All *P. falciparum* positive patients were treated with the AL regimen according to national guidelines and were asked to visit the clinics to evaluate parasite positivity on day 3 of treatment.

### Malaria PCR, genotyping of *kelch13*, *pfcrt*, and *pfmdr1*

Parasite DNA was extracted from one quarter of each blood spot using the QIAamp DNA Blood Mini Kit (QIAGEN, Hilden, Germany). *Plasmodium falciparum* positivity was confirmed by species-specific PCR as previously described [19]. Polymorphisms were determined through direct sequencing, including *kelch13* (propeller domain amino acid positions 427–726), *P. falciparum* chloroquine resistance transporter gene (*pfcrt*; amino acid positions 72–76), and *P. falciparum* multidrug resistance-1 gene (*pfmdr1*; amino acid positions 86, 184, 1034, 1042, and 1246). The detailed protocol of the PCR analysis for each target gene is described in Additional file 1. *Pfcrt* K76T and *pfmdr1* N86Y are associated with chloroquine resistance, and confer chloroquine resistance [20, 21]; the opposite trend has been reported for lumefantrine [22–24]. The allele proportion of *kelch13, pfcrt, *and *pfmdr1* in 2020 was compared to that in 2002, 2003, and 2016–2018 [11, 12, 16]. The genotypes of *kelch13* in 2018 were analysed using the same samples previously reported [12].

### Statistical analysis

Statistical analysis was performed using R software (version 4.1.0), with the Chi-square test for trend (Cochran-Armitage trend test). Statistical significance was set at p < 0.05.

## Results

### Patients and *Plasmodium* sp. specific PCR

**Fig. 1:**
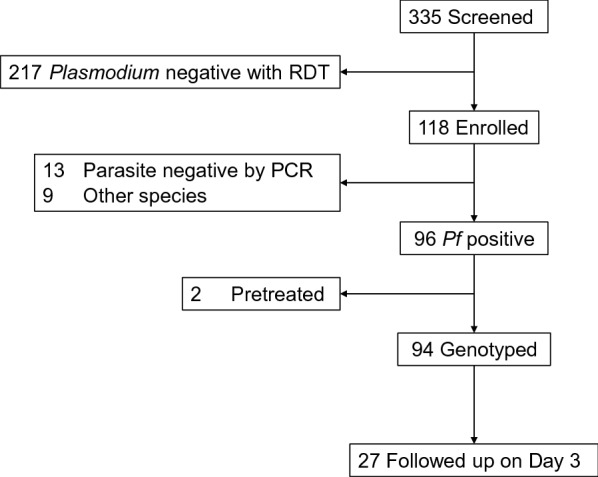
Flow chart of the study

Among the 335 patients screened with RDT, 118 had positive results for *Plasmodium* (Fig. 1). Of these, species-specific PCR revealed that 13 were parasite negative and nine were other species, resulting in 96 patients with *P. falciparum* (Additional file 2). Two patients who received an intramuscular injection of artemether within two weeks prior to enrollment were excluded from further analysis. Finally, 94 samples were used for molecular analysis. There were no significant differences in the background characteristics of the enrolled patients between the two clinics (Table 1). The median age was 17 years old [Inter quartile range (IQR): 13, 26.5, range: 4–7]. The median parasitaemia was2.2 × 10^6^/µl (IQR: 0.9 × 10^6^, 5.6 × 10^6^, range: 0.04 × 10^6^–40.2 × 10^6^). No significant difference was observed between patient number, age, sex, and average parasitaemia in each year (Additional file 3).

**Table 1:** Characteristics of studied patients

Characteristic	Day 1 (n = 94)
Sampling clinics; n (%)	
Wirui	46 (49)
Town	48 (51)
Age (Years); n (%)	
0–9	10 (11)
10–19	41 (44)
20–	43 (46)
Median (IQR*)	17.0 (13.0, 26.5)
Sex; n (%)	
Male	47 (50)
Female	47 (50)
Symptoms; n (%)	
Muscle or joint aches	24 (26)
Chill/Shivering	36 (38)
Headache	60 (64)
Nausea/vomiting	18 (19)
Abdominal pain	14 (15)
Diarrhoea	7 (7)
Cough	24 (26)
Convulsions	8 (9)
Temperature (> 37.5 °C)	47 (50)
Parasitaemia	
Median (Parasites × 10^6^/µl)	2.2
(IQR)	(0.9, 5.6)

^*^IQR Inter Quartile Range

### Frequency of polymorphisms in *kelch13*,* pfcrt*, and *pfmdr1*

**Fig. 2:**
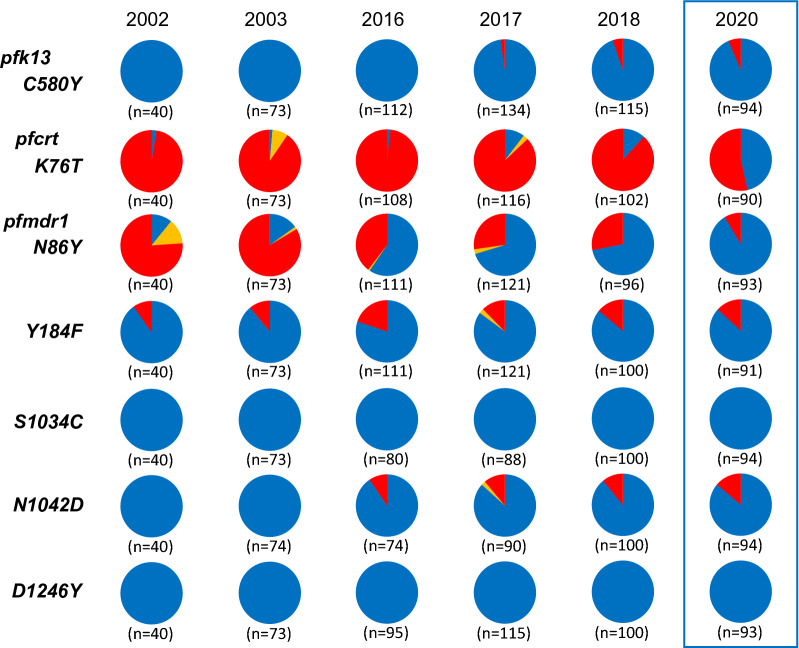
Changes in *kelch13, pfcrt* and *pfmdr1* allele proportion in 2002, 2003, 2016–2018, and 2020. Each colour corresponds an allele type. Blue corresponds to wild-type, yellow to mix, and red to mutant

The frequency of polymorphisms in *kelch13*, *pfcrt*, and *pfmdr1* in 2020 are shown in Fig. 2. C580Y was the only mutation in *kelch13* and was identified in six patients (6.4%). For *pfcrt,* there were two haplotypes, wild-type (CVMNK) and mutant (SVMNT; mutation underlined), with similar proportions (53.3% in wild-type and 46.7% in mutant). In *pfmdr1*, polymorphisms were observed at the amino acid positions 86, 184, and 1042; nearly all harboured the wild-type except the position 184.

These results were compared with the previous results obtained in 2002, 2003, and 2016–2018 (Fig. 2) [11, 12, 16, 17]. All these studies were performed as passive case detections at the same clinics. In *kelch13*, since the first detection of C580Y in three patients (2.2%) in 2017 [11], there has been a statistically significant increase in the proportion of C580Y (p = 4.2 × 10^–3^). In both *pfcrt* and *pfmdr1,* the majority of parasites harboured chloroquine-resistant types in 2002 and 2003 [16]. However, a marked shift of allele proportion to chloroquine-sensitive types was observed after the mid-2010s (Fig. 2). In *pfcrt*, wild-type significantly increased from 1.9% in 2016 to 46.7% in 2020 (p = 8.9 × 10^–16^). In *pfmdr1*, the N86 allele also significantly increased from 59.5% in 2016 to 91.4% in 2020 (p = 2.3 × 10^–6^). The transition to the chloroquine-sensitive form of *pfmdr1* occurred at least three years earlier than that for *pfcrt*. In fact, the proportion of the *pfmdr1*N86 allele in 2016 was higher than that of K76 in *pfcrt* in 2020. Although polymorphisms were observed at positions 184 and 1042, the proportion fluctuated annually without any upward or downward trends. Only the wild-type allele was found throughout the study period (2002–2003, 2016–2018, and 2020) at positions 1034 and 1246*.*

### Follow-up of patients on day 3

Since the patients were asked for a voluntary visit on day 3, among the 94 patients treated with AL, 27 were followed-up with clinical assessment on day 3. Three patients were infected with the C580Y mutant. Only one patient was afebrile on day 3. Prevalent symptoms remaining on day 3 were headache (33%) and muscle/joint pain (15%) (Additional file 4). The smears of all follow-up patients showed an absence of parasites on day 3. There were no cases of early treatment failure.

## Discussion

Ex vivo and molecular epidemiological study related to malaria drug resistance was conducted in 2002 and 2003 in East Sepik, PNG [16, 17]. After a long interval between 2004 and 2015, the study was restarted in 2016 [12]. In 2017, three *kelch13* C580Y mutants were identified [11]. This raised concern that ART-resistant *P. falciparum* parasites may have already emerged and spread in the study area. In this study, the proportion of *kelch13* C580Y was relatively low, but it had increased significantly since the first detection. In general, drug-resistant malaria increases slowly at the beginning of an emergence, but increases rapidly as the frequency of resistant parasites increases. Indeed, C580Y frequencies gradually increased approximately five years from the initial detection, but then rapidly expanded and even overtook the other *kelch13* alleles in Cambodia and Western Thailand [7, 8, 25]. Therefore, even though the current proportion of *kelch13* C580Y is low in PNG, rapid expansion in the near future could be anticipated.

Conversely, regional malaria epidemiological factors in PNG may suppress the rapid increase in ART-resistant parasites. First, residents in the study area developed higher levels of herd immunity to malaria than those in the GMS region because of the higher malaria transmission intensity in PNG [26, 27]. This can considerably influence the clearance of ART-resistant parasites from human hosts [28] and may slow the rate of increase in the C580Y allele in the region. Second, because ART is primarily used in ACT, the presence of resistant parasites to ACT partner drugs significantly affects the diffusion rate of C580Y. In the GMS region, parasites resistant to partner drug(s), mefloquine, and piperaquine have already emerged and spread [29, 30]. In particular, parasites harbouring both *kelch13* C580Y and *plasmepsin* 2/3 copy number variants, which are molecular markers for piperaquine resistance, have rapidly increased in West Cambodia [25, 31]. In contrast, in PNG there is no evidence that parasites are resistant to lumefantrine, the currently used partner drug of ACT. Previous ex vivo drug susceptibility study from 2016 to 2018 also demonstrated that the average IC_50_ to lumefantrine was 4.6 nM and no parasite fulfilled the criteria of ex vivo lumefantrine resistance [12]. Furthermore, no patient exhibited parasite positivity on day 3, although the follow-up number was small.

The effects of the C580Y mutation on ART resistance are important determinants of the survival of drug-resistant parasites. However, the introduction of C580Y into *P. falciparum* clones did not substantially increase the level of in vitro ART resistance compared to other mutations, such as R539T [32]. In addition, drug-resistant mutations generally confer a decrease in parasite fitness, which often leads to a survival disadvantage [33–36]. Several laboratory studies have demonstrated that the growth rates of C580Y harbouring transgenic parasites were equal to or less than those of transgenic parasites with other *kelch13* mutations, such as R561H, E252Q, and G538V. This suggest that C580Y incurs level of fitness impairment that is at least similar to that of the other *kelch13* mutations [32, 37, 38]. These laboratory findings are inconsistent with the field observations in the GMS region, where the C580Y mutant outcompeted other mutants [7, 8, 25]. This implies that some unique background genetic changes in the South-East Asia (SEA) parasites play a beneficial role in the survival of the SEA parasites harbouring the C580Y allele. This might include compensation for the harmful effects of the C580Y mutation [37, 39]. Several single nucleotide polymorphisms have been identified in SEA *kelch13* isolates [40]. However, among thesesingle nucleotide polymorphisms, only one (ferredoxin D193Y) was found in our PNG C580Y mutants [11], suggesting that PNG C580Y mutants do not possess the same background genetic changes as SEA C580Y mutants.

For chloroquine susceptibility, the average IC_50s_ values were still high (80.5–106.6 nM) with a low proportion of *pfcrt* K76 wild-type (2.3–11.7%) during 2016–2018 [12]. In 2020, however, the proportion of *pfcrt* K76 wild-type rapidly increased to 46.7%. Since a significant association between the *pfcrt* K76 wild-type allele and ex vivo chloroquine susceptibility was confirmed [12], the observed rapid increase in the *pfcrt* K76 allele suggests that chloroquine sensitivity has been recovering. The significant increase in *pfmdr1* N86 wild-type from 59.5% to 91.4% in the three years to 2020 also suggests the potential resurgence of chloroquine sensitivity. Although this phenomenon has been widely observed in African countries [13–15], it is very rare in SEA [41].

In the study area, decreased ex vivo susceptibility to lumefantrine was significantly associated with *pfmdr1* N86 [12], consistent with a previous transfection study showing that an allelic change from N86Y to N86 increased IC_50_ for lumefantrine three to four times [24]. There are considerable reports that *pfmdr1* N86 wild-type is selected by AL treatment [22, 23]. Furthermore, a recent meta-analysis of 60 AL clinical trials revealed that only 38% of patients treated with AL were symptomatic when the infection recurred [42]. If patients are asymptomatic at the time of recurrence, they are unlikely to seek treatment, resulting in the persistence of parasitaemia. This would increase the chance of parasites being transmitted to other human hosts, which could subsequently spread the drug-resistant mutations in the parasite population. In addition, a specific *pfmdr1* haplotype (multicopy *pfmdr1* in addition to N86 and Y184F) has increased and become predominant in Cambodia and Vietnam [43]. Gene editing experiments have shown that this haplotype significantly reduces parasite susceptibility to lumefantrine [43]. In the study site, although this haplotype was not detected [11], all these genetic changes (multicopy *pfmdr1*, N86, and Y184F) were individually observed. Therefore, in addition to the assessment of the relapse rate following AL, it is necessary to monitor the appearance of this haplotype.

## Conclusions

Since its first identification in 2017, *kelch13* C580Y harbouring *P. falciparum* parasites have been increasing in Wewak, East Sepik, PNG. A significant increase in *pfcrt* K76 and *pfmdr1* N86 was also observed. This suggests a possible recovery of chloroquine sensitivity and, on the other hand, a decrease in sensitivity to lumefantrine, the ACT partner drug. The increasing frequency of *kelch13* C580Y mutants under these circumstances is a warning sign that parasites resistant to AL will emerge in the near future. Thus, it is important to enhance continuous monitoring to detect early signs of the emergence of ACT-resistant parasites.

## Supplementary Information

**Additional file 1: **Primers and protocol for the PCR analysis.

**Additional file 2:** Molecular diagnosis of enrolled samples.

**Additional file 3:** Background characteristics of enrolled patients each year (2002, 2003, and 2016–2018).

**Additional file 4:** Characteristics of studied patients (Day 3).

## Data Availability

The primary datasets used and analysed during the current study are available from the corresponding author upon reasonable request.

## Abbreviations

ACT: Artemisinin combination therapy
ART: Artemisinin
AL: Artemether plus lumefantrine
GMS: Greater Mekong Subregion
IC_50_: 50% Growth inhibitory concentration
IQR: Inter quartile range
PCR: Polymerase chain reaction
*pfcrt*: *P. falciparum* chloroquine resistance transporter gene
*pfmdr1*: *P. falciparum* multidrug resistance-1 gene
PNG: Papua New Guinea
RDT: Rapid diagnostic test
SEA: Southeast Asia
