# Supplementary material for: Increase in the proportion of Plasmodium falciparum with kelch13 C580Y mutation and decline in pfcrt and pfmdr1 mutant alleles in Papua New Guinea
Source: Malar J. 2021 Oct 19;20:410. doi: 10.1186/s12936-021-03933-6 (PMC8524940; doi:10.1186/s12936-021-03933-6)
Supplement: Supplementary file 1 — Additional file 1: Primers and protocol for the PCR analysis. [file 12936_2021_3933_MOESM1_ESM.pdf]

Primers and protocol for the PCR analysis

Target gene (position) Primer name	Sequence	Annealing Temperature ℃
kelch13 (Propeller domain)		
K13-1R	GGGAATCTGGTGGTAACAGC	60
K13-3end-1R	GGGAAAATCATAAACAATCAAGTAATGTGT	60
K13-2R	GCCTTGTTGAAAGAAGCAGA	60
K13-3end-2R	AATGTGCATGAAAATAAATATTAAAGAAGA	60
pfcrt (72-75)		
Pfcrt-K76T-F1	CGAGCGTTATAGAGAATTAGATAATTTAGT	60
Pfcrt-K76T-R1	GCATCTAACATGGATATAGCAAAAAATTGT	60
Pfcrt-K76T-F2	TTAGGTGGAGGTTCTTGTCTTGGTAAATGT	60
Pfcrt-K76T-F2	GTTTCGGATGTTACAAAACTATAGTTACCA	60
pfmdr1 (86,184)		
Pfmdr A1	TGTTGAAAGATGGGTAAAGAGCAGAAAGA	68
Pfmdr A3	TACTTTCTTATTACATATGACACCACAAACA	68
Pfmdr A4	AAAGATGGTAACCTCAGTATCAAAGAAGAG	65
Pfmdr A2	GTCAAACGTGCATTTTTTATTAATGACCATTTA	65
pfmdr1 (1034,1042,1246)		
Pfmdr O1	AGAAGATTATTTCTGTAATTTGATAGAAAAAGC	45
Pfmdr O2	ATGATTCGATAAATTCATCTATAGCAGCAA	45
Pfmdr 1034f	AGAATTATTGTAAATGCAGCTTTATGGGGACTC	45
Pfmdr 1042r	AATGGATAATATTTCTCAAATGATAACTTAGCA	45

PCR mixture	Volume
TksGflex DNA Polymerase* (1.25 units/μl)	0.1 μl
2× Gflex PCR Buffer (Mg^{2+}, dNTP plus)	5 μl
Template DNA (<500 ng)	1 μl
Primer 1 (5 μM)	0.5 μl
Primer 2 (5 μM)	0.5 μl
DW	up to 10 μl

*TksGflex DNA Polymerase (Takara Bio, Japan)

PCR protocol	Cycles
1) 98°C for 10 s	1
2) 98°C for 10 s<br>Annealing Temperature for 10 sec<br>68°C for 60 s	40<br>(30 for 2nd)
3) 68°C for 3min	1
