# Supplementary material for: Increase in the proportion of Plasmodium falciparum with kelch13 C580Y mutation and decline in pfcrt and pfmdr1 mutant alleles in Papua New Guinea
Source: Malar J. 2021 Oct 19;20:410. doi: 10.1186/s12936-021-03933-6 (PMC8524940; doi:10.1186/s12936-021-03933-6)
Supplement: Supplementary file 2 — Additional file 2: Molecular diagnosis of enrolled samples. [file 12936_2021_3933_MOESM2_ESM.pdf]

Molecular diagnosis of enrolled samples.

| Diagnosis | n=118 |
|---|---|
| *Pf* | 88 |
| *Pv* | 7 |
| *Pf* + *Pv* | 4 |
| *Pf*+ *Pv* + *Po* | 3 |
| *Pf* + *Pm* | 1 |
| *Pv* + *Po* | 2 |
| *Plasmodium* negative | 13 |

*Pf*: *P. falciparum*; *Pv*: *P. vivax*; *Po*: *P. ovale*; *Pm*: *P. malariae.*
