# Supplementary material for: Increase in the proportion of Plasmodium falciparum with kelch13 C580Y mutation and decline in pfcrt and pfmdr1 mutant alleles in Papua New Guinea
Source: Malar J. 2021 Oct 19;20:410. doi: 10.1186/s12936-021-03933-6 (PMC8524940; doi:10.1186/s12936-021-03933-6)
Supplement: Supplementary file 3 — Additional file 3: Background characteristics of enrolled patients each year (2002, 2003, and 2016–2018). [file 12936_2021_3933_MOESM3_ESM.pdf]

Background characteristics of enrolled patients (2002, 2003, and 2016–2018)

Characteristic	2002 (n=40)	2003 (n=73)	2016 (n=123)	2017 (n=134)	2018 (n=111)
Sampling clinics					
Wirui	40	73	89	40	57
Town	0	0	34	94	54
Age (Year)					
Median (IQR)	5 (4.0,7.0)	5 (3.0,7.0)	20 (13.0,33.5)	18 (11.0,24.0)	18 (11.0,26.8)
Sex; n					
Male	26	40	53	57	57
Female	12	33	69	76	53
Unknown	2	0	1	1	1
Parasitaemia (Parasites×10^6/μl)					
Median (IQR)	0.72 (0.36, 1.20)	0.86 (0.32, 2.43)	0.63 (0.09, 2.34)	1.49 (0.45, 3.96)	0.54 (0.45, 2.57)

*IQR=Inter Quartile Range
