# Supplementary material for: Increase in the proportion of Plasmodium falciparum with kelch13 C580Y mutation and decline in pfcrt and pfmdr1 mutant alleles in Papua New Guinea
Source: Malar J. 2021 Oct 19;20:410. doi: 10.1186/s12936-021-03933-6 (PMC8524940; doi:10.1186/s12936-021-03933-6)
Supplement: Supplementary file 4 — Additional file 4: Characteristics of studied patients (Day 3). [file 12936_2021_3933_MOESM4_ESM.pdf]

Characteristics of studied patients (Day 3)

Characteristics	Day 3 (n=27)
Sampling clinics; n (%)	
Wirui	7 (26)
Town	20 (74)
Age (Years); n (%)	
0–9	4 (15)
10–19	7 (26)
20–	16 (59)
Median (*IQR)	25.0 (14.5,33.0)
Sex; n (%)	
Male	12 (44)
Female	15 (56)
Symptoms; n (%)	
Muscle or joint aches	4 (15)
Chill/Shivering	4 (15)
Headache	9 (33)
Nausea/Vomiting	1 (4)
Abdominal pain	0
Diarrhoea	2 (7)
Cough	3 (11)
Convulsions	0 (0)
Temperature (> 37.5°C)	2 (7)
Parasitaemia, Median (Parasites/μl)	0

*IQR=Inter Quartile Range
